# Ag Nanorods Coated with Ultrathin TiO_2_ Shells as Stable and Recyclable SERS Substrates

**DOI:** 10.1038/srep15442

**Published:** 2015-10-21

**Authors:** Lingwei Ma, Yu Huang, Mengjing Hou, Zheng Xie, Zhengjun Zhang

**Affiliations:** 1 State Key Laboratory of New Ceramics and Fine Processing, School of Materials Science and Engineering, Tsinghua University, Beijing 100084, P.R. China; 2 Key Laboratory of Advanced Materials (MOE), School of Materials Science and Engineering, Tsinghua University, Beijing 100084, P.R. China; 3 High-Tech Institute of Xi’an, Shannxi 710025, P.R. China

## Abstract

TiO_2_-coated Ag nanorods (Ag@TiO_2_ NRs) have been fabricated as multifunctional surface-enhanced Raman scattering (SERS) substrates. Uniform TiO_2_ shells could sufficiently protect the internal Ag NRs against oxidation and sulfuration, thus the temporal stability of SERS substrates was markedly improved. Meanwhile, due to the synergetic effect between crystalline TiO_2_ and Ag, the nanocomposites could clean themselves via photocatalytic degradation of the adsorbed molecules under ultraviolet irradiation and water dilution, making the SERS substrates renewable. Such Ag@TiO_2_ NRs were shown to serve as outstanding SERS sensors featuring high sensitivity, superior stability and recyclability.

As an influential spectroscopic detection method for extremely minute amounts of target molecules, surface-enhanced Raman scattering (SERS) technique is currently recognized as one of the most promising analytical tools in fields of chemistry, biology, medicine, and life science^1^,^2^,^3^,^4^, with the advantages of ultrahigh sensitivity and specificity, rapid response speed as well as non-destructive determination^5^,^6^,^7^,^8^. In general, noble metal materials (in particular Au, Ag, and Cu) of multiple nanostructures can render excellent SERS performance, introduced mainly by the electromagnetic (EM) resonance between the incident optical field and localized surface plasmons (LSP)^9^,^10^. In recent years, great efforts have been dedicated to fabricate various noble metal substrates as efficient Raman signal amplifiers and even to meet the requirements for single-molecule detection^11^,^12^,^13^,^14^.

However, from practical application viewpoint, not only strong enhancement but also stability, recyclability as well as cost-effective preparation methods are necessary for satisfactory SERS sensors. To this end, the fabrication of SERS-active substrates with optimized properties is still faced with numerous challenges. For example, despite the optimal plasmonic enhancement of Ag nanostructures^15^,^16^,^17^, their applicability is hindered by the poor stability, which is caused by the oxidation and sulfuration of silver in air^18^,^19^,^20^ and will greatly weaken the SERS performance. Furthermore, traditional SERS substrates with noble metals are not easily reused, thus the high costs would seriously hamper the universality of SERS technique^21^,^22^,^23^. For these reasons, it is significant to develop stable and recyclable SERS substrates. Lately, nanocomposites consisting of noble metals and photocatalytic materials have been elaborately designed and synthesized, which showed great potential to satisfy the above demands^12^,^24^,^25^,^26^,^27^,^28^.

Herein, we reported the preparation of Ag nanorods coated with uniform TiO_2_ overlayers (Ag@TiO_2_ NRs), for the synthesis of sensitive, stable, and recyclable SERS substrates. Slanted Ag NRs were prepared based on oblique angle vapor deposition (OAD)^29^,^30^,^31^ technique, and were subsequently covered with TiO_2_ layers using atomic layer deposition (ALD)^19^,^32^. In order to achieve the recyclability of the substrates, further thermal annealing at 300 °C was employed to improve the crystallinity of TiO_2_, which was crucial for photocatalytic activity. The Raman enhancement originates from ordered Ag NRs, and TiO_2_ shells function as a barrier that could protect the internal Ag NRs at elevated temperatures as well as avoid their direct contact with external environments. What’s more, the self-cleaning ability stems from the ultraviolet (UV) light-induced degradation of analytes via the synergetic effect of TiO_2_ shells and Ag NRs, i.e., subsequent to SERS measurements, the substrates can be purified by UV irradiation and be reused for further Raman analyses. This self-cleaning function offers an opportunity to eliminate the single-use shortcoming of most conventional SERS substrates and reduce the SERS measurement costs as well.

## Results and Discussion

### Characterization of Ag@TiO_2_ NRs

**Figure 1:**
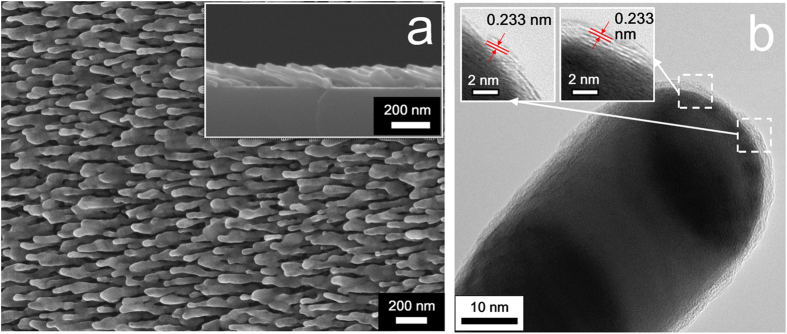
(**a**) Top-view and side-view SEM images of Ag@TiO_2_-3 NRs. (**b**) HRTEM images of a Ag@TiO_2_-3 NR after annealing at 300 °C.

Ag NRs coated with TiO_2_ shells by 1, 2, 3, 4, and 5 ALD cycles are denoted hereafter as Ag@TiO_2_-1, Ag@TiO_2_-2, Ag@TiO_2_-3, Ag@TiO_2_-4, and Ag@TiO_2_-5, respectively. Figure 1a shows the typical top-view and side-view SEM images of Ag@TiO_2_-3 NRs. It could be observed that the tilted NRs are ~40 nm in diameter, ~280 nm in length, and well-separated. Note that, due to the relative low-temperature ALD procedure (at 80 °C) and the generated TiO_2_ shells, there were no evident fusion and distortion of the underneath Ag NRs during ALD coating and the subsequent annealing treatments^19^,^33^,^34^. In addition, we found that the substrates coated by different ALD cycles showed no visible structure variation observed from the SEM resolution, which might be due to the ultrathin nature of the deposited TiO_2_ layers.

We thus used TEM analyses to provide a visual evidence of the TiO_2_ coatings, which were also applied to probe the thickness growth of TiO_2_ layers. The TEM images of Ag@TiO_2_ substrates coated with 1 to 5 ALD cycles are displayed in Fig. S1. It is shown that the TiO_2_ coatings grown at 80 °C are extremely conformal and uniform with varying thickness, fully wrapping the Ag NRs, and are amorphous in structure. A linear relationship between TiO_2_ thickness and ALD cycles was obtained at ~0.55 nm per cycle, which was ascribed to the intrinsic characteristic of ALD process on the basis of sequential self-terminating growth^32^. Further, after annealing at 300 °C, Ag@TiO_2_-3 NRs exhibit the lattice spacing of 0.233 nm obtained from HRTEM image in Fig. 1b. This lattice spacing corresponds to the distance between the (112) crystal planes of anatase^35^,^36^ and indicates the crystallization of TiO_2_ at high temperature.

**Figure 2:**
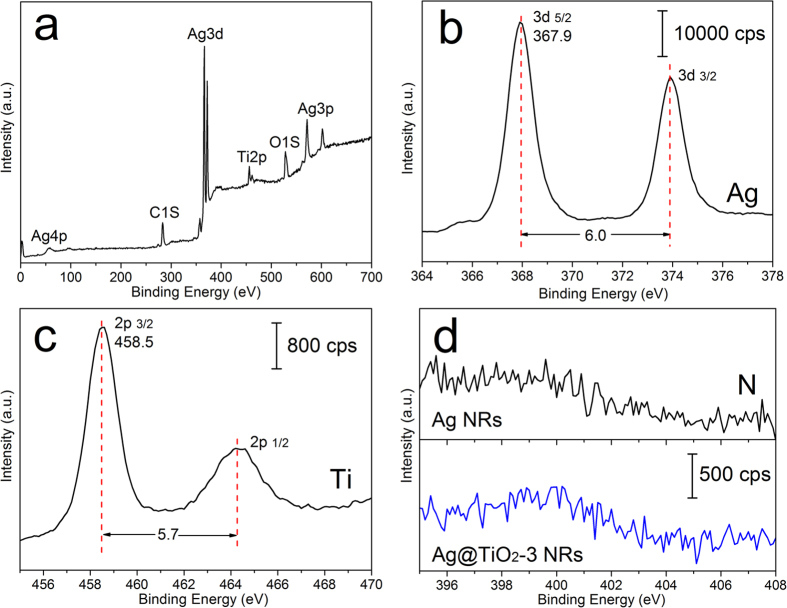
(**a**) XPS survey spectrum taken from Ag@TiO_2_-3 NRs. (**b**) HRXPS Ag 3d spectrum on Ag@TiO_2_-3 NRs. (**c**) HRXPS Ti 2p spectrum on Ag@TiO_2_-3 NRs. (**d**) HRXPS N 1 s spectra on different substrates: the spectrum above is from bare Ag NRs, and the one below is from Ag@TiO_2_-3 NRs. All binding energies of the XPS spectra are calibrated with reference to the C1s peak at 284.8 eV.

XPS measurements were conducted to further verify the surface components and valence states of Ag@TiO_2_ substrates. As shown in Fig. 2a, no excessive peaks other than Ag, Ti, O, and C signals are observed from Ag@TiO_2_-3 NRs. Figure 2b plots the XPS spectrum of Ag 3d_5/2_ and Ag 3d_3/2_ double peaks from the substrate, which are centered at 367.8 and 373.8 eV, respectively. This is in good agreement with those of elemental Ag^37^,^38^, and demonstrates that there was no corrosion of Ag NRs during ALD and post-annealing procedures. Figure 2c shows the amplified Ti 2p_3/2_ and Ti 2p_1/2_ XPS peaks at 458.5 and 464.2 eV, indicating the formation of Ti^4+^ in TiO_2_ 39,40. In addition, compared with TiO_2_ molecules, the ALD precursor tetrakis(dimethylamino)titanium (TDMAT) contains four N atoms in each molecule^41^, so the XPS spectrum of N element could be used to further evaluate the reaction extent of TDMAT and water. Figure 2d represents the XPS spectra of N 1 s from bare Ag and Ag@TiO_2_-3 NRs. No visible peaks were observed in both curves, which means that the Ti-N bonds of TDMAT were fully broken and the chemical reaction during ALD process was complete.

### Sensitivity and Stability of Ag@TiO_2_ NRs

**Figure 3:**
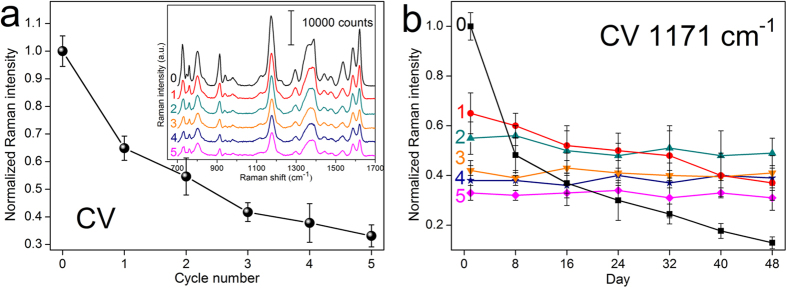
(**a**) The normalized Raman intensities of 1171 cm^−1^ peak from 10^−5^ M CV molecules versus the ALD cycles of Ag@TiO_2_ NRs. The inset illustrates the Raman spectra of 10^−5^ M CV adsorbed on bare Ag NRs and Ag@TiO_2_-1, Ag@TiO_2_-2, Ag@TiO_2_-3, Ag@TiO_2_-4, Ag@TiO_2_-5 NRs, respectively. (**b**) The normalized Raman intensities of 1171 cm^−1^ peak on these substrates during aging in air for 48 days.

It has been long recognized that the Raman enhancement of metal nanostructures depends strongly on the distance between metal surfaces and adsorbed molecules^19^,^42^,^43^. We therefore investigated the TiO_2_ layers’ effect on the sensitivity of SERS substrates, using crystal violet (CV) as a model analyte. The inset in Fig. 3a shows the SERS spectra of 10^−5^ M CV on bare Ag NRs and Ag NRs coated with TiO_2_ layers by 1 to 5 ALD cycles. It is observed that all spectra with distinct intensities clearly reveal several characteristic Raman bands of CV molecules^44^. Herein, the 1171 cm^−1^ Raman peak with strong intensity was chosen to quantitatively calculate the attenuation effect of TiO_2_ layers on SERS sensitivity. Figure 3a plots the normalized Raman intensities at 1171 cm^−1^ as a function of ALD cycles. As expect, due to the enhancive analyte-substrate separation introduced by TiO_2_, CV Raman signals decreased monotonously with the increase of ALD cycles. To be specific, the CV intensities on Ag@TiO_2_-1, Ag@TiO_2_-2, and Ag@TiO_2_-3 substrates occupied ~65%, ~55%, and ~43%, respectively, compared with those on bare Ag NRs, and then decreased mildly when further increasing the TiO_2_ thickness. It is noted that, although the Raman enhancement dropped to some extent when TiO_2_ became thicker, all coated substrates still exhibited large Raman signals with little background noises. Fig. S2 shows the Raman spectra of CV on Ag@TiO_2_-2 NRs at concentrations ranging from 10^−5^ M to 10^−10^ M. One sees that the Raman intensities descend along with CV concentrations, nevertheless, their Raman peaks were readily observed even when the concentration was as low as 10^−9^ M. Meanwhile, the detection limits for Ag@TiO_2_-3 and Ag@TiO_2_-4 NRs were down to 10^−8^ M (spectra not shown here), demonstrating the superior sensitivity of Ag@TiO_2_ nanocomposites.

The temporal stability of bare Ag and Ag@TiO_2_ NRs was supervised via counting the SERS intensities of 10^−5^ M CV from the stored substrates as a function of time. Figure 3b displays the normalized Raman intensities at 1171 cm^−1^ band obtained from different substrates over a period of 48 days. For each sample, the 1171 cm^−1^ peak value was normalized to that on freshly prepared Ag NRs to facilitate comparison. As can been seen, CV signals on bare Ag NRs emerged a substantial decline even only after 8 days (~50% drop), and were more than one order smaller after 48 days. However, when the NRs were uniformly wrapped with ultrathin TiO_2_, their stability was dramatically enhanced. Specifically, Ag@TiO_2_-1 NRs presented a slight decrease in SERS performance, while the substrates covered with TiO_2_ by two or more cycles remained unchanged in SERS enhancement during the whole test period. As a result, the TiO_2_ shells could protect the internal Ag NRs against at atmospheric conditions, which were valuable in improving the corresponding stability of SERS substrates.

### Recyclability of Ag@TiO_2_ NRs Substrates

**Figure 4:**
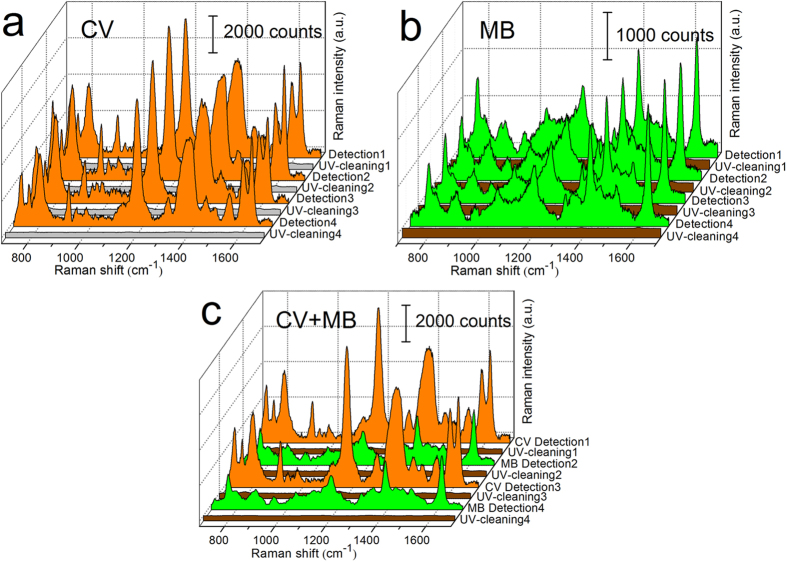
Raman spectra of (**a**) 10^−5^ M CV, (**b**) 5 × 10^−6^ M MB, and (**c**) CV and MB alternately adsorbed onto Ag@TiO_2_-3 NRs in four “detection-UV cleaning” cycles. Each cycle consists of the adsorption of target molecules followed by UV irradiation. The graphs show the Raman spectra before and after self-cleaning.

In addition to sensitivity and stability, the recyclable property of SERS substrates is also meaningful for routine applications^19^,^43^,^45^. In our study, the self-cleaning performances of Ag@TiO_2_ NRs were investigated through UV light-assisted photocatalytic bleaching of organic molecules adsorbed on the substrates. These experiments were performed by the following setups: after SERS characterization of the analytes adsorbed on Ag@TiO_2_ NRs, the substrates were immersed into deionized water and were irradiated by UV light for certain times. When the organic molecules were completely decomposed, these substrates could be reused for further analyte immersion and SERS measurements. The primary results showed that the Ag NRs coated with TiO_2_ layers by 3, 4, and 5 cycles could degrade most of the CV molecules absorbed on them through UV radiation within 20 minutes, while Ag NRs with thinner TiO_2_ films could not achieve an effective decomposition of dye molecules within a short time. Hence, Ag@TiO_2_-3 NRs were chosen to verify the UV-assisted renewability, due to their relatively high SERS sensitivity compared with that of Ag@TiO_2_-4 and Ag@TiO_2_-5 substrates. As shown in Fig. 4a, four circulations of the “detection-UV cleaning” process were carried out. Strong Raman signals of 10^−5^ M CV were observed in the first round, while no obvious CV peaks were identified after UV-illumination, suggesting that the target molecules were gradually decomposed into small inorganic species such as CO_2_, HCl, and H_2_O that could be removed easily by aqueous solvent^26^. In this way, this substrate could be reused as a new and clean SERS sensor, while it was not possible to wash the adsorbed molecules off the substrate barely by water (see Fig. S3). More importantly, the results from the subsequent three circulations showed that the Raman intensities of CV maintained almost at the same level in every detection step, which indicates that the Ag@TiO_2_ structure could endure multiple UV irradiations, enabling it to work as a reversible SERS substrate with high robustness.

In order to further confirm the universality of this multifunctional substrate, 5 × 10^−6^ M methylene blue (MB) molecules were introduced to perform the “detection-UV cleaning” routes. As shown in Fig. S4, intense MB Raman signals are carried out on Ag NRs covered with various cycles of TiO_2_. Typically, there was also a descending relationship between Raman intensities and TiO_2_ cycles, which was in accord with the results we reached before. The Ag@TiO_2_-3 substrate was employed again in the reusability tests. Figure 4b shows the Raman spectra of MB on the substrate before and after self-cleaning. One sees clearly that the MB signals almost vanished after 30-minute UV illumination, and were fully recovered through subsequent MB soaking. Last but not least, different molecules can also be alternately examined and degraded over the same substrate. As shown in Fig. 4c, the representative vibration patterns of CV and MB were both clearly identified when dyes were adsorbed on Ag@TiO_2_-3 NRs, but were completely disappeared via UV irradiation and water dilution. We should note that the measurement of different molecules did not influence each other since only the Raman peaks of the adsorptive molecules were discovered other than any impurity peaks. These results suggest that the Ag@TiO_2_ composites were feasible to act as recyclable SERS substrates for the detection of organic species such as CV and MB, and show great potential for further sensitive and reusable SERS applications^13^,^26^,^28^.

For comparison, this photocatalysis process was carried out on bare Ag NRs under the identical experiment conditions. From Fig. S5a, one sees that after UV irradiation for 30 minutes, the Raman peaks of MB were still clearly observed on Ag NRs, and the decline of Raman signals may owing to the molecule dilution in water. Moreover, in the following three “detection-UV cleaning” cycles, when the substrate was further soaked into MB solution, the Raman signals dropped gradually time after time. Fig. S5b,c present the SEM images of Ag NRs adsorbed with MB, before and after UV irradiation for 60 minutes. Apparent coarsening was observed from the illuminated Ag NRs, i.e., the high power UV radiation gave rise to a detrimental effect on bare Ag NRs, resulting in the decrease in SERS sensitivity.

### Photocatalytic Mechanism for Ag@TiO_2_ NRs

**Figure 5:**
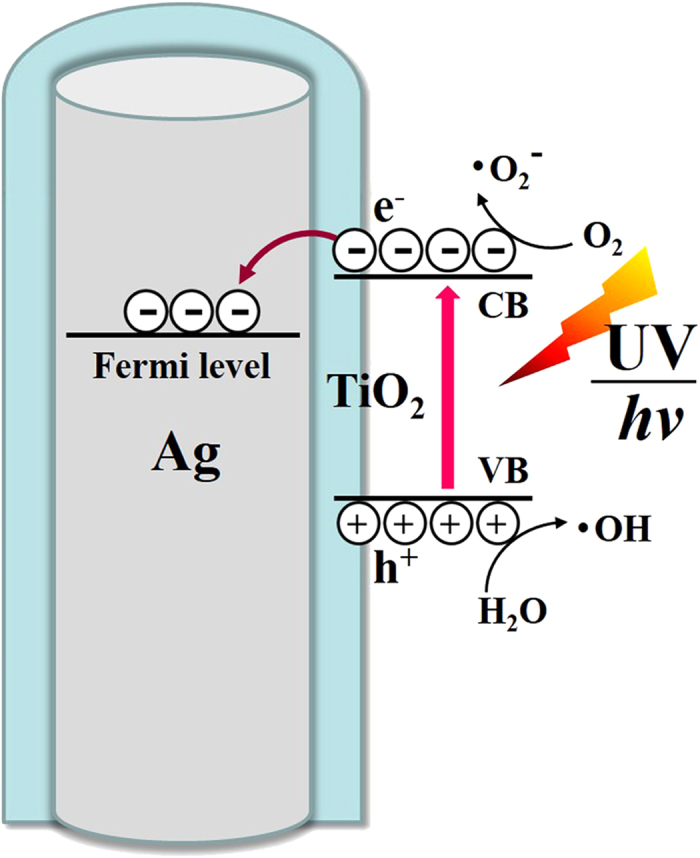
The schematic for the photocatalytic mechanism of Ag@TiO_2_ NRs.

In the area of semiconductor-noble metal SERS substrates, TiO_2_ has attracted considerable attention owing to its strong chemical stability, remarkable photocatalytic activity, and low-cost synthesis^46^,^47^,^48^. It is commonly accepted that the photocatalytic ability of TiO_2_ depends greatly on its crystal types, and anatase is usually more active than rutile and amorphous TiO_2_ 49,50,51. We thus annealed the Ag@TiO_2_ NRs at 300 °C to crystalize the amorphous TiO_2_ to anatase, in order to boost their photocatalytic performance. Additionally, Ag NRs in contact with TiO_2_ layers can act as electron traps to separate the photogenerated electron-hole pairs (e^−^ − h^+^), which is beneficial for photocatalytic reactions^13^,^52^,^53^. Figure 5 illustrates the mechanism of such a process. When TiO_2_ is activated by UV light, it undergoes charge separation and the e^−^ are excited into the conduction band of TiO_2_. Since the work function of Ag is lower than that of TiO_2_ 51,53, Ag serves as an acceptor of the e^−^ transformed from TiO_2_ layers, while the h^+^ remain in TiO_2_. As a consequence, Ag@TiO_2_ NRs provide a charge transfer channel at the metal-semiconductor interface, and Ag could extensively suppress the recombination of e^−^ − h^+^ pairs. The residual e^−^ on the surface of TiO_2_ can be trapped via O_2_ to form superoxide (•O_2_^−^)^54^, while h^+^ at the valence band of TiO_2_ are oxidized by H_2_O to form surface hydroxyl radical (•OH)^55^. Thereafter, the organic molecules nearby will react with these active oxidative species and be decomposed into inorganic compounds. In this way, the strong interaction between Ag and TiO_2_ would optimize the separation of photo-excited charge carriers, resulting in a remarkable enhancement of organic molecules degradation efficiency.

## Conclusion

In summary, we have developed a facile and novel approach for the fabrication of Ag NRs coated with uniform TiO_2_ shells as sensitive, stable, and reusable SERS substrates, and found that Ag NRs covered by ~2 nm TiO_2_ shell was optimal in our study. TiO_2_ overlayers could protect Ag NRs against the disturbance from air, without seriously weakening the sensitivity of SERS substrates. Moreover, due to the beneficial interaction between Ag NRs and TiO_2_ layers, dye molecules adsorbed on Ag@TiO_2_ NRs were rapidly photodegraded into inorganic species under UV irradiation, thus the renewed substrate could be used for further SERS analyses with little decline in sensitivity. This study suggests that Ag@TiO_2_ nanocomposites with stability and self-cleaning property can serve as superb substrates in SERS sensing fields.

## Methods

### Fabrication of Ag NRs

Slanted Ag NRs were grown on Si (001) substrates by OAD technique in an electron-beam system (GLAD, Thermionics Inc.) with a background vacuum level down to 10^−6^ Pa. During deposition, the angle between the surface normal of substrates and the incoming vapor flux was set at ~86°, and the deposition rate as well as thickness were simultaneously monitored via a quartz crystal microbalance (QCM). The growth rate was fixed at ~0.75 nm/s, and the deposition stopped when the QCM read 500 nm. The detailed deposition procedure can be found elsewhere^56^,^57^,^58^.

### Fabrication of Ag@TiO_2_ NRs

TiO_2_ adhesion layers were deposited onto freshly prepared Ag NRs in an ALD reactor (MNT-100, Wuxi MNT Micro and Nanotech Co.). The TiO_2_ precursors, i.e., TDMAT (maintained at 110 °C) and water (maintained at 40 °C) were alternately pumped through the reaction chamber, using high purity N_2_ (99.999%, 15 sccm) as the carrier and purge gas. The chamber was heated and maintained at 80 °C so as to guarantee a complete chemical reaction of precursors, while not damaging the morphology of Ag NRs at the relatively low reaction temperature. Typically, one complete reaction cycle took ~38 s and consisted of four steps: (1) TDMAT reactant was pulsed for 200 ms and allowed to soak in an additional 5 s; (2) N_2_ gas was used to purge the chamber for 20 s; (3) water vapor was pulsed for 6 ms and soaked for an extra 3 s; and (4) the chamber was purified by N_2_ for 6 s. This reaction cycle repeated for 1, 2, 3, 4, and 5 times over Ag NRs, and the as-prepared samples were further annealed at 300 °C for 30 minutes in a quartz tube furnace in air.

### Characterization

The morphology, structure, and chemical states of Ag NRs and Ag@TiO_2_ NRs were characterized by scanning electron microscope (SEM, JEOL-JMS-7001F), high-resolution transmission electron microscope (HRTEM, JEOL-2011) and X-ray photoelectron spectroscopy (XPS, PHI 5300) with Mg Kα as the excitation source, respectively.

### Measurements of SERS Sensitivity and Recyclability

The SERS performances were evaluated by an optical fiber micro-Raman system (i-Raman Plus, B&W TEK Inc.) using CV and MB as probing molecules. Before SERS measurements, all substrates were submerged into dye aqueous solutions for 30 minutes, and dried naturally in air. The Raman spectra were obtained using a 785 nm laser as the excitation source, with its beam spot focused to ~80 μm in diameter and an excitation power of 120 mW. The integration time of one spectrum was 15 s and 6 s for CV and MB, separately. For every sample, the Raman spectrum was obtained by averaging the spectra obtained from five different areas of the SERS substrate.

To test the UV-cleanable property, the photocatalytic self-cleaning experiments were performed by the following setups: after SERS characterization of the organic molecules adsorbed on Ag@TiO_2_ and bare Ag NRs (bare Ag NRs were used in a control experiment), these substrates were immersed into a vessel containing 50 mL deionized water and were irradiated by a 300 W Xe lamp for certain times. An ultraviolet cutoff filter was inserted between the light source and the substrates to block the UV light with the wavelength below 420 nm. The electric current was set at 15 A and the power density of the UV light was ~60 mW/cm^2^. Water was used to accelerate the dilution effect and get rid of the thermal energy coming from UV radiation. Then the illuminated substrates were dried in air and the following Raman spectra were executed to check the degradation degree of adsorbates. Finally, when the substrates showed no apparent Raman signals, they could be reused for further SERS trials. For the reusability characterization, the “detection-UV cleaning” cycles were repeated for four times on each sample.

## Additional Information

**How to cite this article**: Ma, L. *et al.* Ag Nanorods Coated with Ultrathin TiO_2_ Shells as Stable and Recyclable SERS Substrates. *Sci. Rep.* **5**, 15442; doi: 10.1038/srep15442 (2015).

## Supplementary Material

Supplementary Information
