# Supplementary material for: Ag Nanorods Coated with Ultrathin TiO2 Shells as Stable and Recyclable SERS Substrates
Source: Sci Rep. 2015 Oct 21;5:15442. doi: 10.1038/srep15442 (PMC4614262; doi:10.1038/srep15442)
Supplement: Supplementary Information [file srep15442-s1.pdf]

Supporting Information

Ag Nanorods Coated with Ultrathin TiO_2 Shells as Stable and Recyclable SERS Substrates

Lingwei Ma[1], Yu Huang[1], Mengjing Hou[1], Zheng Xie[3], and Zhengjun Zhang[2]*

[1] State Key Laboratory of New Ceramics and Fine Processing, School of Materials Science and Engineering, Tsinghua University, Beijing 100084, P.R. China,

[2] Key Laboratory of Advanced Materials (MOE), School of Materials Science and Engineering, Tsinghua University, Beijing 100084, P.R. China.

[3] High-Tech Institute of Xi'an, Shannxi 710025, P.R. China

* Author to whom all correspondence should be addressed.

* E-mail: zjzhang@tsinghua.edu.cn.

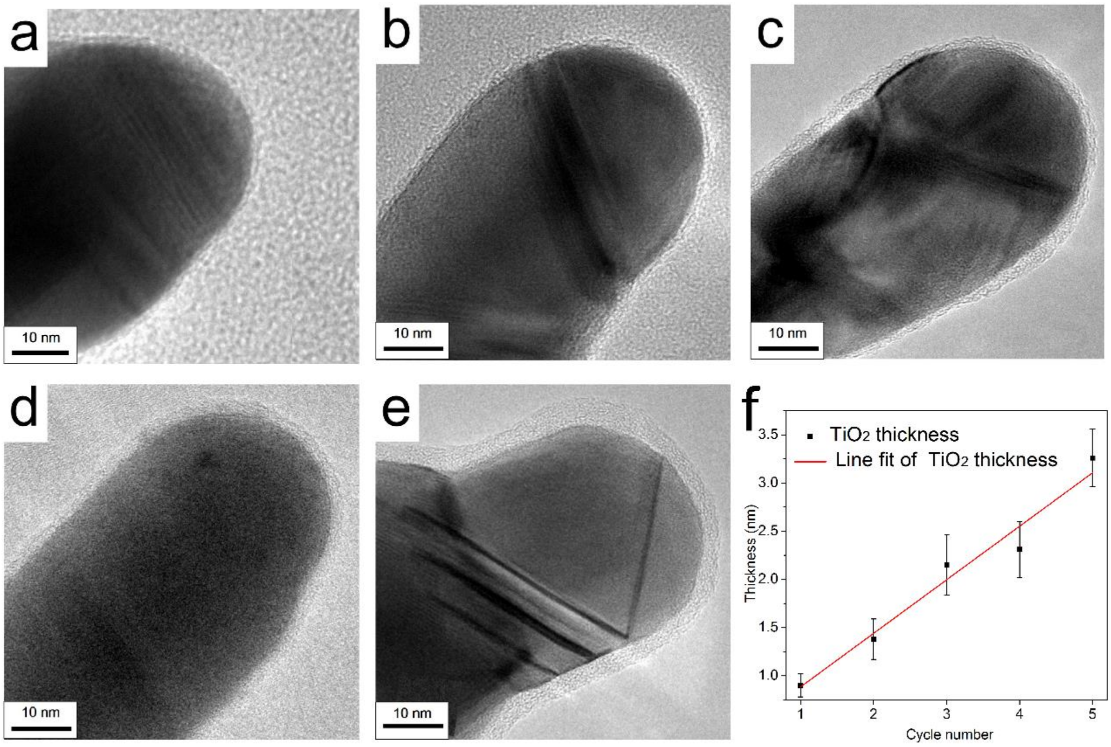

Figure S1. TEM micrographs of Ag NRs following (a) 1, (b) 2, (c) 3, (d) 4 and (e) 5 ALD cycles of TiO_2 at 80 ℃. (f) The plots of average TiO_2 thickness as a function of growth cycles and the corresponding linear regression. In each picture, the TiO_2 thickness was calculated by averaging the layer thickness measured at ten positions picked randomly along Ag NRs.

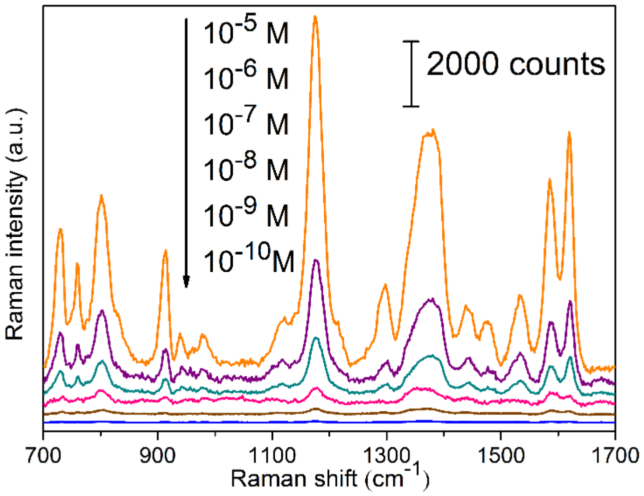

Figure S2. Raman spectra of 10^{-5} M to 10^{-10} M CV molecules adsorbed on Ag@TiO_2-2 NRs.

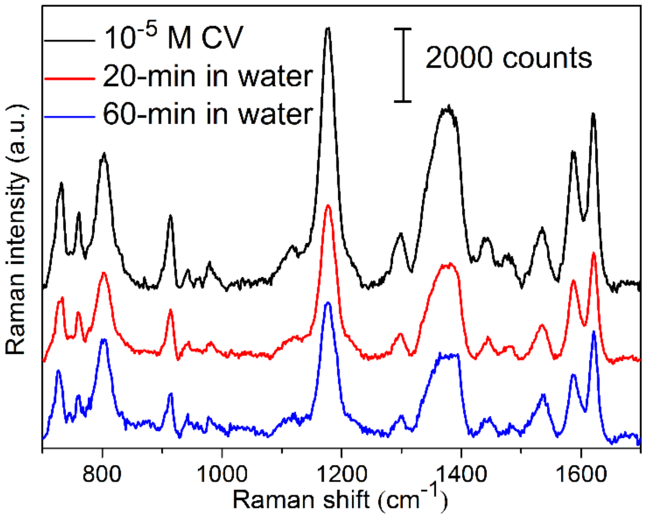

Figure S3. Raman spectra of 10^{-5} M CV molecules adsorbed on Ag@TiO_2-3 NRs (a) before and after (b) 20-min and (c) 60-min water dilution.

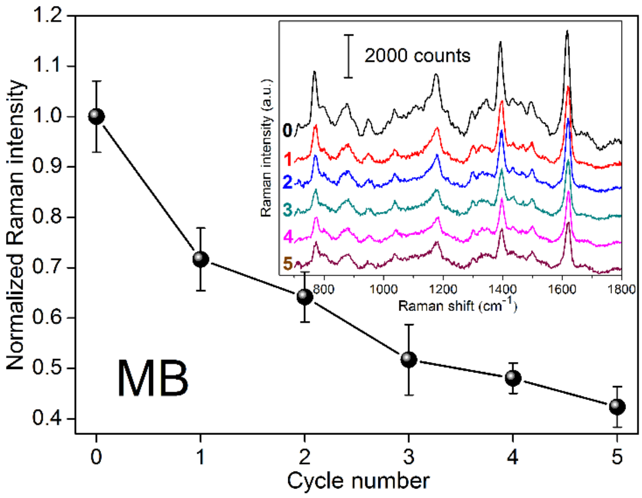

Figure S4. The normalized Raman intensities of 1622 cm^{-1} peak from 5×10^{-6} M MB molecules versus the ALD cycles of Ag@TiO_2 NRs. The inset illustrates the Raman spectra of MB adsorbed on bare Ag NRs and Ag@TiO_2-1, Ag@TiO_2-2, Ag@TiO_2-3, Ag@TiO_2-4, Ag@TiO_2-5 NRs, respectively.

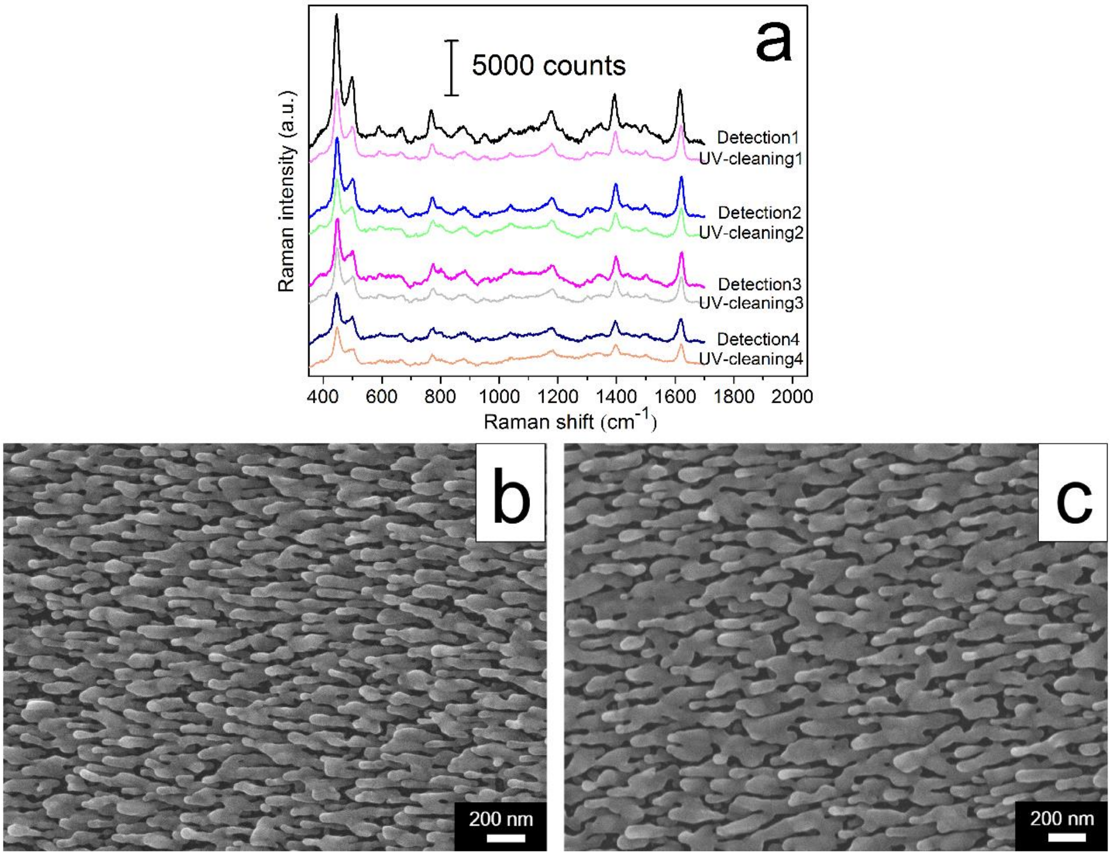

Figure S5. (a) Raman spectra of 5×10^{-6} M MB adsorbed onto bare Ag NRs with four "detection-UV cleaning" cycles. SEM images of bare Ag NRs adsorbed with MB (b) before and (c) after being irradiated by UV light for 60 minutes.
